# Public Health Stops at the School House Door

**DOI:** 10.1289/EHP530

**Published:** 2016-10-01

**Authors:** Jerome A. Paulson, Claire L. Barnett

**Affiliations:** 1 Pediatrics and Environmental and Occupational Health, George Washington University School of Medicine and Health Science, and; 2 Milken Institute School of Public Health, George Washington University, Washington, D.C., USA; 3 Healthy Schools Network, Inc., Albany, New York, USA

## Abstract

In the United States, all children of appropriate age are required to attend school, and many parents send their children to child care. Many school and day care buildings have been found to have environmental health problems that impact children’s health and diminish their ability to learn. No federal agency has the capacity or authority to identify, track, or remediate these problems. A recent meeting, coordinated by Healthy Schools Network, Inc., has developed a set of recommendations to begin to deal with the issue of environmental health problems in schools.

## Introduction

Children are required by law to attend school in the United States; many parents voluntarily send their children to preschool or child care centers.

Environmental health threats in child care centers and in pre-kindergarten to 12th grade (PK–12) schools compromise children’s health and learning; yet there is no federal, state, or local agency that is authorized, funded, and staffed to protect children in these settings from environmental health hazards.

## Discussion

### Lack of Data and Data Sharing Hampers Children’s Health Protection

There is no systematic collection of environmental health data on children attending child care or PK–12 schools by any state or federal environmental, health, or education agencies (Paulson and Barnett 2010). Without timely, accurate information, child health and facility health issues cannot be identified or tracked, improvements cannot be documented, and appropriate policy cannot be formulated.

The Family Educational Rights and Privacy Act of 1974 (FERPA 2000) governs data collected by school employees, which can include health data from school nurses or other school employees (ASTHO 2012). The FERPA restrictions make data sharing more difficult than even the Health Insurance Portability and Accountability Act of 1996 (HIPAA 1996).

### School Building Environmental Hazards

Environmental health hazards in schools have been documented in the media in a number of places in the United States (Martin 2012; Stevens 2013; Zaniewski 2016; Purcell and Graham 2013).

Many school buildings in the United States are old and in poor condition. Recent data indicate that 53% of reported schools need to do repairs, renovations, or modernization to bring buildings into good condition. In addition, environmental factors were rated unsatisfactory or very unsatisfactory in 5–17% of permanent buildings and 10–28% in portable buildings (Alexander and Lewis 2014).

All buildings can have a myriad of indoor and outdoor environmental problems (Table 1).

**Table 1:** Potential environmental health problems in schools.

Indoor	Outdoor
Toxic debris from construction or renovation in occupied building	Use of lawn chemicals, including pesticides
Infiltration of air pollution from outside air or ground—nearby industry, construction on site or near by, nearby transportation corridors	Artificial turf
Noise from inside or outside	Allergens
Air pollution from indoor construction equipment, paints, glues, new carpets, etc.	Schools located on toxic sites (Brownfields, National Priority List sites)
Air pollution from occupants—third-hand tobacco, wood smoke, dry cleaning chemicals, personal care products	Toxic debris from construction or renovation
Water damage, dampness leading to growing molds and other substances	Air pollution from nearby industry, construction on site or near by, nearby transportation corridors
Excess CO_2_ from inadequate ventilation	Bus and vehicle idling at school
Inadequate lighting	Vermin, pests
Allergens—from in-school vermin, air infiltration, transported in on clothing, school pets, or service animals
Chemical exposures—lab chemicals, cleaning supplies, pesticides, educational supplies; copiers, vocational, and other education supplies
Radon
Asbestos
Polychlorinated biphenyls in lighting ballast, caulk, floors, and ceiling tiles
Lead in paint or water
Inadequate heating or cooling
Note: Adapted from the Center for Health, Environment, and Justice, “Poisoned Schools: Invisible Threats, Visible Actions,“ https//www.chej.org/publications/health.htm. Agency for Toxic Substances and Disease Registry with U.S. EPA and Morehouse School of Medicine Regional Research Center for Minority Health (oral presentation at American Public Health Association, October 2001).	

### Lack of Legislation and Regulation

There are few laws or regulations governing indoor environmental health or other aspects of environmental health in schools (see Environmental Law Institute, http://www.eli.org/buildings/topics-school-environmental-health-overview-state-laws and http://www.eli.org/buildings/database-state-indoor-air-quality-laws). Therefore, many environmental problems are unaddressed or left to voluntary programs, many of which were established by the U.S. Environmental Protection Agency (EPA) (Table 2); however, the U.S. EPA has had significant budget cuts, which have affected these and other programs.

**Table 2:** U.S. EPA documents providing guidance on environmental health in schools.

U.S. EPA Product	Content	URL
IAQ Tools For Schools Action Kit	Recommendations for managing IAQ, including radon, molds, cleaning, inspections	http://www.epa.gov/iaq-schools
IAQ Design Tools For Schools	Designing new buildings with IAQ in mind	http://www.epa.gov/iaq-schools
HealthySEAT (version 2)	Comprehensive recommendations for schools, including all federal regulations, customizable by states and districts	http://epaschools-stage.icfwebservices.com/guidelinestools/healthySEAT/basic.html
Voluntary Guidelines for States: Development and Implementation of a School Environmental Health Program	Help states develop or expand environmental health programs for K–12 schools	http://www.epa.gov/schools/state-school-environmental-health-guidelines
Programs to reduce exposure to diesel exhaust from school buses	Address the issue of pollutants from diesel school buses	http://www.epa.gov/schools-transportation/schools-school-buses
School siting guidelines	Provide information on how to evaluate environmental factors to make the best possible school siting decisions	http://www.epa.gov/schools/school-siting-guidelines
Toolkit for Safe Chemical Management	Provides information to start or improve a program to reduce chemical hazards and prevent future chemical mismanagement issues	http://www.epa.gov/schools-chemicals
Drinking Water at Schools	Focuses on lead and copper in drinking water	http://www.epa.gov/schools-air-water-quality/schools-water-quality

### Do Green Buildings or Good Environments Support Health and Academic Success?

A National Research Council (NRC) committee concluded that six factors support child and teacher health, learning, and productivity: a dry building with good indoor air quality (IAQ) and thermal comfort that is quiet, clean, and well maintained (Committee to Review and Assess the Health and Productivity Benefits of Green Schools 2007). Another committee concluded that conventional “green buildings” may not protect human health (IOM 2011).

Excess moisture can lead to mold and bacterial growth and degrade building materials. Some of the chemicals released as a result are allergens, irritants, and toxins (Committee on Damp Indoor Spaces and Health 2004). As documented by Purcell and Graham (2013), the presence of these chemicals in the air are associated with multiple health symptoms and complaints as well as short- and long-term health problems among occupants.

Research has shown that poor IAQ has negative impacts on children’s performance in school. The recommended ventilation rate is 15 cubic feet per minute (ANSI/ASHRAE 2013); but many schools do not meet the recommendation (Shendell et al. 2004a; Jenkins et al. 2004; Shaughnessy et al. 2006). Studies have demonstrated that reaction times were faster and speed of schoolwork tasks improved in classrooms with higher ventilation rates (Myhrvold and Olesen 1997; Wargocki and Wyon 2007a, 2007b; Annesi-Maesano et al. 2012); and standardized test scores increased with improved ventilation rates (Haverinen-Shaughnessy et al. 2011). Other studies have shown that higher CO_2_ levels have been associated with decreased cognitive function as measured by standard progressive matrices (Hutter et al. 2013). Mendell et al. (2013) demonstrated that improved ventilation rates also led to a 1–2% decrease in absentee rates. Research by others indicates that some of the decreased absenteeism is related to a decrease in asthma attacks and other respiratory symptoms (Annesi-Maesano et al. 2012; Lai et al. 2015).

Children attending schools located near transportation corridors, air pollution–emitting industries, and other sources of outdoor air pollution are likely to be exposed to those outdoor pollutants while at school (U.S. EPA 2016b; Godoi et al. 2013; Rivas et al. 2014). Indoor air levels of many pollutants may be 2–5 times, and occasionally, > 100 times higher than outdoor levels (U.S. EPA 2016a).

Noise in classrooms may interfere with learning. Various studies have shown that in noisy classrooms children may have difficulty comprehending spoken information, and several studies have shown that academic achievement and behavior are compromised (Shendell et al. 2004b; Clark and Sörqvist 2012). Other studies have shown specifically that a 5-decibel difference in aircraft noise coming into the classroom was equivalent to a 2-month reading delay in the United Kingdom (Stansfeld et al. 2005) and a 1-month reading delay in the Netherlands (van Kempen et al. 2010). These studies also demonstrated adverse impacts on recognition, memory, and annoyance. Other research indicates that noise interferes more with complex tasks than simpler tasks (van Kempen et al. 2010).

Early 20th-century schools were often built with very large windows allowing for natural light and ventilation. Later in the century, school buildings were constructed with smaller, or occasionally nonexistent, windows as an energy saving measure. However, research indicates that children achieve better test scores and exhibit better behavior with controlled day lighting combined with appropriate artificial lighting (Committee to Review and Assess the Health and Productivity Benefits of Green Schools 2007; Edwards and Torcellini 2002). In other studies, controlled variation of lighting showed a 16.8% improvement in words read. Reading comprehension also improved, but the results were not statistically significant (Barkmann et al. 2012).

Thermal comfort is a combination of air temperature, radiant temperature, relative humidity, and air speed (Purcell and Graham 2013). Based on limited evidence about children and more robust evidence about adults in office buildings, the NRC concluded that thermal comfort is important to academic performance (Purcell and Graham 2013). Studies of academic performance in the temperature range of 20–25°C showed variable results (Wargocki and Wyon 2007a; Haverinen-Shaughnessy and Shaughnessy 2015).

The notion of a well-maintained and clean school incorporates multiple actions and building systems: for example, pest control and pesticide use, and “green cleaning.” There is growing evidence that long-term, low-dose pesticide exposure at certain times of life leads to adverse outcomes (Rosas and Eskenazi 2008; González-Alzaga 2014). While little of this research pertains to schools or school-age children, it is prudent to limit pesticide use in schools and on school grounds. About two-thirds of states have some legislation or regulation related to reducing pesticide use in schools and about half of those require integrated pest management, a pesticides-last approach (National Association of State Boards of Education 2013).

A well-accepted definition of green cleaning products has emerged from International Organization for Standardization (ISO)-compliant third-party certifiers with standards for sensitive populations (Barnett 2013). These standards ban or steeply restrict phthalates, asthmagens, carcinogens, reproductive toxins, and certain sensitizers. Currently, 11 states and the District of Columbia have adopted policies requiring or promoting green cleaning in schools; California and Massachusetts state-operated asthma programs also promote green cleaning in schools (Coalition for Healthier Schools 2015). Research is needed to assess how these products impact school attendance, achievement, and productivity.

### Green Buildings Do Not Assure Child (or Adult) Health

There is no single accepted definition of a green building or a green school building (Committee to Review and Assess the Health and Productivity Benefits of Green Schools 2007). There are at least four sets of differing design standards for green or high-performance school buildings. The U.S. Green Building Council (USGBC) has standards for green buildings known as Leadership in Energy and Environmental Design (LEED) (USGBC 2014). The U.S. EPA’s voluntary IAQ Design Tools for Schools provides strategies for school construction and renovation issues (U.S. EPA 2015). The U.S. Department of Energy’s (DOE) National Best Practices Manual for Building High Performance Schools promotes energy efficiency and renewable energy (U.S. DOE 2007). The Collaborative for High Performance Schools has standards (http://www.chps.net/dev/Drupal/node/212) that began with LEED with the goal of addressing educational and indoor environmental quality. The WELL Building Standard® (WELL) is a relatively new program that establishes criteria for building and renovation that are specifically directed at factors that affect health. While promising, there are no specific criteria related to schools, and the program is too new to have outcome measures at this time (International WELL Building Institute 2015). Although there are no peer-reviewed studies documenting the health benefits of conventional green schools as of 2012 (Worden et al. 2014), 24 states had either voluntary or required advanced school design standards (Coalition for Healthier Schools 2015).

## Conclusions

### Recommendations from the Meeting’s Participants

In November 2015, Healthy Schools Network, Inc. convened the first national facilitated discussion of children’s environmental health in schools and child care centers (Healthy Schools Network 2015). The authors organized the meeting that consisted of a public forum with presentations from the U.S. EPA, the National Institute of Occupational Safety and Health (NIOSH), and indoor air researchers; a facilitated workshop was also organized and attended by representatives of more than two dozen federal and state health agencies, as well as an array of nongovernment organizations (NGOs) from the fields of health, environment, and education. While the meeting was not facilitated to full consensus, the authors, advised by the NGO attendees, summarized the following major recommendations from official notes taken during the meeting:

***Call to Action.*** There are scores of national organizations concerned about traditional school health (Michael et al. 2015), but this effort is distinct from the traditional view and should be known as “Environmental Health at School,” as the panel and workshop are titled.

***Develop a communication and advocacy strategy.*** Advocacy organizations should coordinate a communication and advocacy strategy to demonstrate the urgent moral, ethical, cost savings, and legal imperatives to care for children where they learn and play and to integrate children’s environmental health into public health and into education. The message should be that environmental health considerations must be prioritized when siting, designing, constructing, renovating, and maintaining educational facilities. In addition, educational personnel and officials should receive training in environmental health topics relevant to schools and child care facilities. To support these efforts, a national network of stakeholders should be created to engage champions in states and localities, leverage Congressional support through personal testimony, and develop white papers for the incoming presidential administration to encourage policy reform.

***Implement necessary legislative and regulatory changes.*** The federal government could develop minimum standards for protecting children’s environmental health in schools and child care facilities. Simultaneously, advocates could explore mechanisms for adapting elements of the NIOSH and the Occupational Safety and Health Administration (OSHA) programs that were established for worker health and safety to help set up an independent system to protect children in schools and in child care facilities. In addition, the Centers for Disease Control and Prevention (CDC) could strengthen the coordination of its programs to ensure that issues related to children’s environmental health in schools and child care facilities are adequately addressed and prioritized. Changes in state policies may also be needed.

***Healthy Children, Healthy Schools’ reports.*** High-level reports could be commissioned to review existing information on children’s environmental health in schools and child care facilities and provide recommendations on actions related to children’s environmental health in schools. The National Academies of Science, Engineering, and Medicine or the President’s Task Force on Environmental Health and Safety Risks could produce these reports. The topics could include reviewing the existing literature, developing a study of the scale of children’s environmental health needs in schools and child care facilities, and identifying prevention and mitigation strategies for primary and secondary environmental health risks to children in these venues.

***Establish a National Healthy Children, Healthy Schools Commission.*** The commission could be created by the federal government and charged with following up on any recommendations developed as a result of special reports on key topics, such as developing research, collecting and managing data, and posting the results of school assessments and registered complaints. The commission would be a public–private partnership and should include the U.S. EPA, the National Center for Environmental Health (NCEH), the Agency for Toxic Substances and Disease Registry (ATSDR), the NIOSH, the National Institute of Environmental Health Sciences, the U.S. Department of Education, NGOs, and knowledgeable parents and community-based advocates.

***Responding to the Civil and Disability Rights challenges.*** An analysis of how the Department of Education’s Office for Civil Rights has handled environmental health issues in accommodation requests as well as a legal analysis of school and or state agency liability for children’s environmental health at school should be addressed. Another report should analyze if and how federal disability laws and regulations could protect children impacted by environmental factors.

***Develop effective facility prevention programs across the country.*** There could be a two-tiered approach to inspections. School districts could conduct maintenance, monitoring of identified risk factors, and inspections. To accomplish this, a committee of school nurses, facilities staff, and parents, or an independent, state-licensed third party could conduct regular walk-throughs. A regulatory authority such as state or local health departments could conduct routine regulatory inspections to assess environmental health and safety conditions in schools and child care facilities. The CDC’s School Health Policies and Practices Survey (SHPPS) could be improved to collect and report trends in the environmental quality in PK–12 facilities. However, the survey would need significant expansion to address child care.

***Develop institutional tools or mechanisms for identifying at-risk children.*** Tools or mechanisms could be developed to ensure that at-risk children receive appropriate assistance and to improve the identification of those who are medically fragile. This includes using syndromic surveillance to receive chief complaints.

***Develop effective prevention and intervention systems for children across the country.*** To establish effective intervention systems across the country, an independent program similar to the efforts of the NIOSH and OSHA models could be developed to cover children in their workplaces (i.e., schools and child care facilities). In addition, the Pediatric Environmental Health Specialty Units (PEHSUs) and/or state health departments could receive complaints about environmental exposures at schools and child care facilities and work with state and local health departments to conduct onsite investigations. To expand and support an effective intervention program, state-specific handbooks of state regulations and the rights of disabled children could be developed as a desktop reference for addressing children’s school-based risks and exposures.

***Conduct pilot studies of the proposed prevention, intervention, and tracking programs.*** The Council of State and Territorial Epidemiologists and/or other entities could conduct pilot studies for tracking and surveillance programs.

***Establish training, education, and guidance programs for parents and guardians, teachers and principals, health care providers, and public health professionals.*** PEHSUs, federal agencies, educational unions or associations, local and state health departments, and ATSDR regional representatives could develop training programs and materials that are tailored to each stakeholder group. These trainings should include general information about the kinds of environmental risks and exposures found in schools and specific concerns for sensitive populations, as well as guidance on *a*) harmful practices that contribute to the persistence of environmental risks and exposures in schools, *b*) how to recognize problems, *c*) how to take an environmental health history, and *d*) whom to contact in case of an emergency.

Responding to the Call for Action to improve children’s health in schools and protect them from environmental health threats, several steps have already begun and a follow-on meeting is scheduled for June 2016. The plan is to have the attendees work to describe how a National Healthy Children, Healthy Schools Commission might be established, funded, and operationalized. The issue of the intersection between disability rights and environmental health in schools will be further explored. The authors and the attendees of the November 2015 meeting hope that additional exploration of topics covered in this meeting may reveal leverage points for decreasing environmental health threats to children in schools. Attendees will work to develop mechanisms for identifying children at risk from environmental health hazards in schools and devise recommendations for tracking and monitoring systems. It is expected that these discussions will result in executable plans for pilot projects that can be funded and implemented.
